# Effect of gestational diabetes mellitus on pregnancy outcomes among younger and older women and its additive interaction with advanced maternal age

**DOI:** 10.3389/fendo.2023.1158969

**Published:** 2023-05-10

**Authors:** Jiangheng Li, Jingli Yan, Linghua Ma, Yongquan Huang, Maoling Zhu, Wu Jiang

**Affiliations:** Department of Maternity-Child Health and Family Planning Services, Nanning Maternal and Child Health Hospital, Nanning, China

**Keywords:** gestational diabetes mellitus, advanced maternal age, pregnancy outcomes, additive interaction, polyhydramnios, preeclampsia

## Abstract

**Background:**

The prevalence of gestational diabetes mellitus (GDM) and advanced maternal age (AMA, ≥ 35 years) has shown an increasing trend worldwide. This study aimed to evaluate the risk of pregnancy outcomes among younger (20-34 years) and older (≥ 35 years) women with GDM and further analyze the epidemiologic interaction of GDM and AMA on these outcomes.

**Methods:**

This historical cohort study included 105 683 singleton pregnant women aged 20 years or older between January 2012 and December 2015 in China. Stratified by maternal age, the associations between GDM and pregnancy outcomes were analyzed by performing logistic regression. Epidemiologic interactions were assessed by using relative excess risk due to interaction (RERI), attributable proportion due to interaction (AP), and synergy index (SI) with their 95% confidence intervals (95%CIs).

**Results:**

Among younger women, individuals with GDM had a higher risk of all maternal outcomes, preterm birth (relative risk [RR] 1.67, 95%CI 1.50–1.85), low birthweight (RR 1.24, 95%CI 1.09–1.41), large for gestational age (RR 1.51, 95%CI 1.40–1.63), macrosomia (RR 1.54, 95%CI 1.31–1.79), and fetal distress (RR 1.56, 95%CI 1.37–1.77) than those without GDM. Among older women, GDM increased the risk of gestational hypertension (RR 2.17, 95%CI 1.65–2.83), preeclampsia (RR 2.30, 95%CI 1.81–2.93), polyhydramnios (RR 3.46, 95%CI 2.01–5.96), cesarean delivery (RR 1.18, 95%CI 1.10–1.25), preterm birth (RR 1.35, 95%CI 1.14–1.60), large for gestational age (RR 1.40, 95%CI 1.23–1.60), macrosomia (RR 1.65, 95%CI 1.28–2.14) and fetal distress (RR 1.46, 95%CI 1.12–1.90). Additive interactions of GDM and AMA on polyhydramnios and preeclampsia were found, with RERI of 3.11 (95%CI 0.05-6.16) and 1.43 (95%CI 0.09-2.77), AP of 0.51 (95%CI 0.22-0.80) and 0.27 (95%CI 0.07-0.46), and SI of 2.59 (95%CI 1.17-5.77) and 1.49 (95%CI 1.07-2.07), respectively.

**Conclusion:**

GDM is an independent risk factor for multiple adverse pregnancy outcomes, and may exert additive interactions with AMA on the risk of polyhydramnios and preeclampsia.

## Introduction

Gestational diabetes mellitus (GDM), a state of hyperglycemia that is first recognized during pregnancy, has an overall incidence of 14 cases per 100 persons globally per year, and its regional standardized prevalence ranges from 7.1% in the Caribbean and North America to 27.6% in North Africa and the Middle East (1). The prevalence of GDM was 14.8% in mainland China, varying from 2.3% to 24.2% in different regions, and has been dramatically increasing over the past decades (2–5). GDM has caused an enormous health and economic burden in China. Population-based studies demonstrated that GDM was associated with an elevated risk of adverse outcomes for mothers and their infants (6, 7). For example, GDM may increase the risk of cesarean delivery, gestational hypertension, and preeclampsia for the mothers, as well as the risk of fetal distress, preterm birth, and macrosomia for their infants (6, 8–10).

The associations of GDM with pregnancy outcomes may change by maternal age. A historical cohort study of 8844 singleton pregnancies observed that GDM elevated the risk of preterm birth and macrosomia among women aged < 35 years, while the increased risk for the two outcomes was not found in women aged 35 years or older (8). A registry-based study in Finland showed that the risk of preterm birth was increased in younger women with insulin-treated GDM but did not rise in older women affected by GDM (11). However, linear relationships between fasting plasma glucose and the risk of preterm birth and macrosomia in both maternal age groups were also demonstrated in a population-based study from Southern China (12). Therefore, it is necessary to further evaluated the associations between GDM and pregnancy outcomes stratified by maternal age.

In the past decades, the proportion of advanced maternal age (AMA, ≥ 35 years) has elevated rapidly, not only in developed countries, but also in some developing countries, including China (13). An increasing number of studies have suggested that pregnant women with AMA were at a higher risk of adverse pregnancy outcomes (14–16). To our knowledge, the separate effect of GDM or AMA on pregnancy outcomes has been well-studied; however, few studies have yet documented the combined impact of GDM and AMA on these outcomes. Our objective was to assess the individual or combined effects of GDM and AMA on pregnancy outcomes using a historical cohort study. This investigation would help us comprehensively estimate the risk of adverse outcomes among pregnant individuals with both GDM and AMA.

## Methods

### Study design and participants

**Figure 1:**
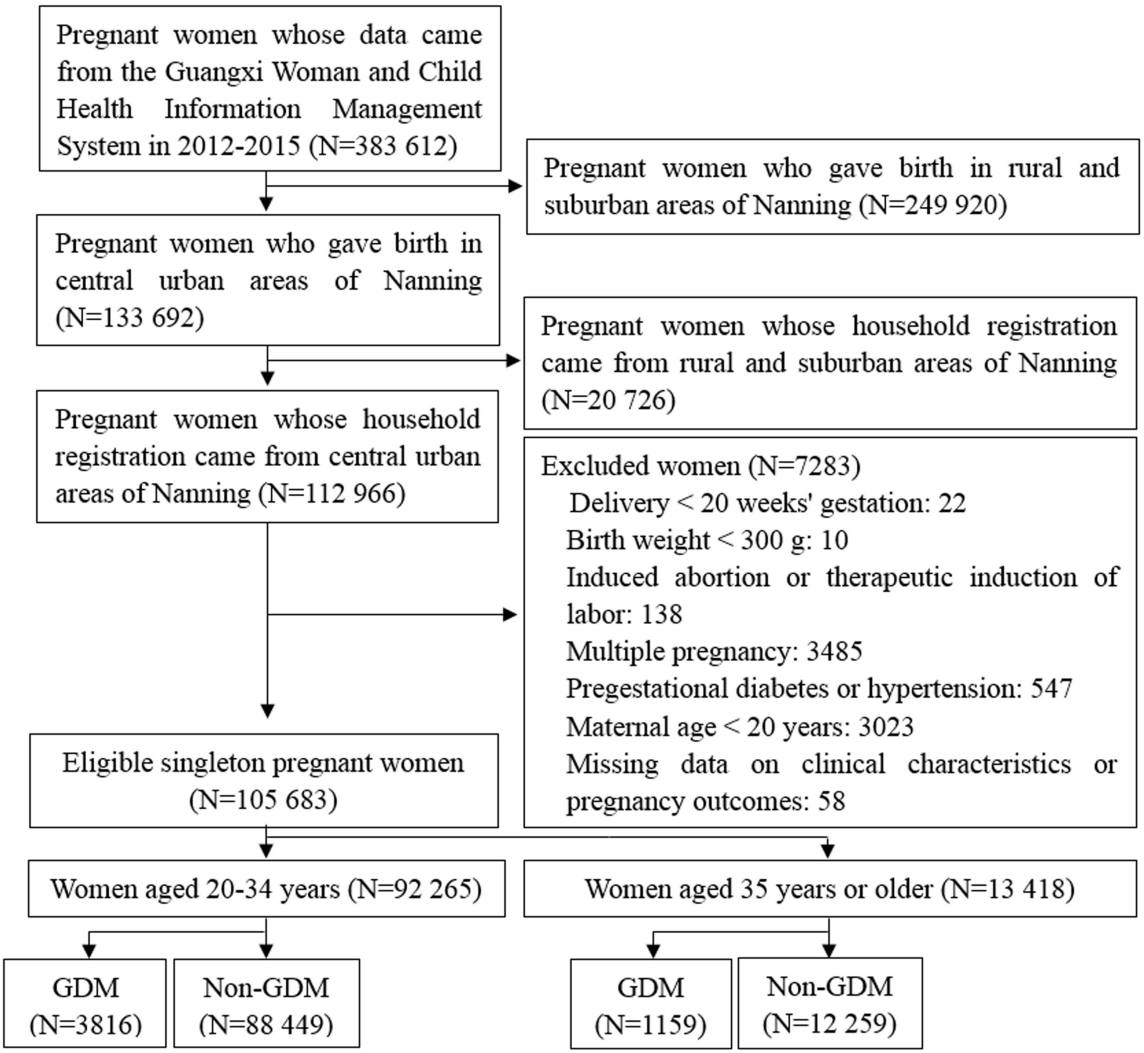
Study flow diagram. GDM, gestational diabetes mellitus.

A historical cohort study of pregnant women aged 20–54 years old was conducted in 27 hospitals located in central urban areas of Nanning, Guangxi province, from January 2012 to December 2015. All studied population derived from a universal GDM screening. Participants were categorized as younger (20-34 years) and older (≥ 35 years) women. We further allocated each of them to a group with GDM and a group without GDM according to the results of 75 g oral glucose tolerance test. Individuals with pregestational diabetes or hypertension, multiple pregnancy, induced abortion, delivery before 20 weeks of gestation, and birthweight less than 300 g were excluded. Study flow chart is shown in **Figure 1**.

This study was approved by the Ethics Committee of Nanning Maternal and Child Health Hospital.

### Data collection

The clinical characteristics and pregnancy outcomes data were collected retrospectively from the Guangxi Woman and Child Health Information Management System. With the guidelines and regulations of the Guangxi Health Commission, all eligible hospitals in Nanning were required to extract information about antenatal care, delivery and infant outcomes from the medical records and input them into this provincial database system. The data entry and management methods were implemented per the previous study (17). Clinical characteristics assessed were: gravidity, parity, obesity (pre-pregnancy body mass index ≥ 30 kg/m^2^), examination at first trimester, number of prenatal visits, previous cesarean history, prior spontaneous or induced abortion and assisted reproductive technology (ART).

### Variables and definitions

We defined GDM as fasting plasma glucose ≥ 5.1 mmol/l or the 75 g oral glucose tolerance test value ≥ 10.0 mmol/l at 60 min or ≥ 8.5 mmol/l at 120 min when conducted at 24-28 gestational weeks (18). AMA was defined as being 35 years or older at the time of giving birth. Pregnancy outcomes included maternal outcomes and infant outcomes.

For maternal outcomes variables, gestational hypertension was diagnosed by blood pressure (BP) monitoring performed after 20 gestational weeks, with a systolic BP ≥ 140 mmHg or a diastolic BP ≥ 90 mmHg. Gestational hypertension with proteinuria was diagnosed as preeclampsia (19). Placental abruption referred to a part or all of the placenta separation from the uterine wall after 20 weeks of gestation (20). Placenta previa referred to a state where the placenta partially or completely covered the opening of the cervix (21). Polyhydramnios was defined as an amniotic fluid volume of over 2000 ml when giving birth. Cesarean delivery referred to a way of giving birth through abdominal and uterine incision.

For infant outcomes variables, we defined small for gestational age (SGA) as a birthweight less than the 10th percentile for its gestational age, large for gestational age (LGA) as greater than the 90th percentile, preterm birth as less than 37 weeks of pregnancy, macrosomia as a birthweight not less than 4000 g, low birthweight as less than 2500 g, low Apgar score as the score at 5 min less than 7, respectively. Fetal distress referred to a syndrome in which the fetus was suffering from insufficient oxygen supply (22). Auricle malformation or external auditory canal atresia was diagnosed as congenital microtia.

### Statistical analysis

Pearson’s chi-square test was used to compare the distributions of clinical characteristics and pregnancy outcomes among two groups, stratified by maternal age. Logistic regression models were implemented to explore the associations between GDM, AMA and adverse pregnancy outcomes.

Given that the odds ratio (OR) always overestimates the relative risk (RR) and it does not have as intuitive an interpretation as the RR (23), we decided to use RR with a 95% confidence interval (95% CI) to assess the association between two categorical variables. RR was calculated by using a formula: RR = OR/[(1 - P_0_) + (P_0_ × OR)], and P_0_ refers to the incidence of the outcome of interest in the reference group (24). Epidemiologic interactions between GDM and AMA on the risk of adverse pregnancy outcomes were assessed via the relative excess risk due to interaction (RERI), attributable proportion due to interaction (AP), and synergy index (SI). The RERI, AP, and SI were separately defined as follows: RERI = RR_11_ − RR_10_ − RR_01_ + 1, AP = RERI/RR_11_, and SI = [RR_11_ − 1]/[(RR_10_ − 1) + (RR_01_ − 1)], where RR_11_, RR_10_, and RR_01_ represented the RR with both GDM and AMA, with GDM only, and with AMA only, respectively. No additive interaction was defined as 95% CI of RERI and AP including 0 and 95% CI of SI comprising 1. The 95% CIs for RERI, AP, and SI were calculated using the method of Hosmer et al. (25) and Andersson et al. (26). The binary and categorical variables were presented as numbers (percentage). All statistical analyses were performed using SPSS version 17.0 (SPSS, Chicago, IL, USA). *P* < 0.05 was considered statistical significance.

## Results

### Population characteristics of the participants

In total, 105 683 singleton pregnancies of women aged 20 years or older were included in our study. The prevalence of GDM was 4.71%, and the proportion of AMA was 12.70%. As depicted in **Table 1**, the two groups were comparable in term of gravidity (*P* > 0.05), but differed in both younger and older women with regard to parity, obesity, examination at first trimester, number of prenatal visits, previous cesarean history, ART, and number of prior spontaneous or induced abortions (all *P* < 0.001). Pregnant individuals with GDM were more likely to have higher proportions of primiparity, obesity, examination at first trimester, prenatal visits ≥ 5 times, previous cesarean history, ART, and prior spontaneous or induced abortion ≥ 3 times when compared to those with normal glucose level regardless of maternal age (all *P* < 0.001).

**Table 1:** Baseline characteristics of women with gestational diabetes mellitus, stratified by maternal age.

Characteristics	Total (n=105 683)	20-34 Years			35 Years or Older		
		GDM (n=3816)	Non-GDM (n=88 449)	*P*-value	GDM (n=1159)	Non-GDM (n=12 259)	*P*-value
Obesity				<0.001			<0.001
No	105 055 (99.41)	3742 (98.06)	88 051 (99.55)		1126 (97.15)	12 136 (99.00)	
Yes	628 (0.59)	74 (1.94)	398 (0.45)		33 (2.85)	123 (1.00)	
Examination at first trimester				<0.001			<0.001
No	45 673 (43.22)	1456 (38.16)	38 090 (43.06)		426 (36.76)	5701 (46.50)	
Yes	60 010 (56.78)	2360 (61.84)	50 359 (56.94)		733 (63.24)	6558 (53.50)	
Number of prenatal visits				<0.001			<0.001
0-4	69 087 (65.37)	2321 (60.82)	57 421 (64.92)		719 (62.04)	8626 (70.36)	
5 or More	36 596 (34.63)	1495 (39.18)	31 028 (35.08)		440 (37.96)	3633 (29.64)	
Gravidity				0.774			0.955
1-2	68 573 (64.89)	2625 (68.79)	61 038 (69.01)		425 (36.67)	4485 (36.59)	
3 or More	37 110 (35.11)	1191 (31.21)	27 411 (30.99)		734 (63.33)	7774 (63.41)	
Parity				<0.001			<0.001
Nulliparous	57 522 (54.43)	2608 (60.60)	51 390 (54.12)		407 (35.12)	3117 (25.43)	
Parous	48 161 (45.57)	1208 (39.40)	37 059 (45.88)		752 (64.88)	9142 (74.57)	
Previous caesarean history				<0.001			<0.001
No	99 655 (94.30)	3485 (91.33)	84 424 (95.45)		912 (78.69)	10 834 (88.38)	
Yes	6028 (5.70)	331 (8.67)	4025 (4.55)		247 (21.31)	1425 (11.62)	
Prior spontaneous or induced abortion				<0.001			<0.001
0-1	100 297 (94.90)	3531 (92.53)	84 742 (95.81)		950 (81.97)	11 074 (90.33)	
2	3586 (3.39)	200 (5.24)	2572 (2.91)		110 (9.49)	704 (5.74)	
3 or More	1800 (1.70)	85 (2.23)	1135 (1.28)		99 (8.54)	481 (3.92)	
ART				<0.001			<0.001
No	104 972 (99.33)	3764 (98.64)	88 025 (99.52)		1109 (95.69)	12 074 (98.49)	
Yes	711 (0.67)	52 (1.36)	424(0.48)		50 (4.31)	185 (1.51)	

GDM, gestational diabetes mellitus; ART, assisted reproductive technology.

Data are n (%) unless otherwise specified.

### Prevalence of pregnancy outcomes among younger and older women with gestational diabetes mellitus

The incidence of pregnancy outcomes among younger and older women with GDM is manifested in **Table 2**. For younger women, the prevalence of all the selected maternal outcomes, preterm birth, low birthweight, LGA, macrosomia, and fetal distress was significantly higher in individuals with GDM than those without GDM (all *P* < 0.001). For older women, individuals with GDM were at a greater incidence of gestational hypertension, preeclampsia, polyhydramnios, cesarean delivery, preterm birth, low birthweight, LGA, macrosomia, and fetal distress compared with those who did not have GDM (all *P* < 0.05). However, a lower prevalence of SGA was observed in individuals with GDM compared to individuals with normal glucose level regardless of maternal age (*P* < 0.05).

**Table 2:** Prevalence of adverse pregnancy outcomes in women with gestational diabetes mellitus, stratified by maternal age.

Outcomes	Total (n=105 683)	20-34 Years			35 Years or Older		
		GDM (n=3816)	Non-GDM (n=88 449)	*P*-value	GDM (n=1159)	Non-GDM (n=12 259)	*P*-value
Gestational hypertension	1281 (1.21)	126 (3.30)	806 (0.91)	<0.001	65 (5.61)	284 (2.32)	<0.001
Preeclampsia	1697 (1.61)	137 (3.59)	1142 (1.29)	<0.001	79 (6.82)	339 (2.77)	<0.001
Placental abruption	338 (0.32)	23 (0.60)	247 (0.28)	<0.001	7 (0.60)	61 (0.50)	0.626
Placenta previa	1046 (0.99)	60 (1.57)	716 (0.81)	<0.001	27 (2.33)	243 (1.98)	0.421
Polyhydramnios	308 (0.29)	21 (0.55)	216 (0.24)	<0.001	18 (1.55)	53 (0.43)	<0.001
Cesarean delivery	34 133 (32.30)	1658 (43.45)	25 885 (29.27)	<0.001	726 (62.64)	5864 (47.83)	<0.001
Preterm birth	6400 (6.06)	352 (9.22)	4840 (5.47)	<0.001	139 (11.99)	1069 (8.72)	<0.001
Low birthweight	5835 (5.52)	252 (6.60)	4606 (5.21)	<0.001	101 (8.71)	876 (7.15)	0.049
SGA	9772 (9.25)	283 (7.42)	8394 (9.49)	<0.001	76 (6.56)	1019 (8.31)	0.037
LGA	11268(10.66)	567(14.86)	8853(10.01)	<0.001	212(18.29)	1636(13.35)	<0.001
Macrosomia	3209 (3.04)	167 (4.38)	2545 (2.88)	<0.001	65 (5.61)	432 (3.52)	<0.001
Fetal distress	3982 (3.77)	241 (6.32)	3245 (3.67)	<0.001	64 (5.52)	432 (3.52)	<0.001
Apgar score < 7 at 5 min	764 (0.72)	19 (0.50)	604 (0.68)	0.172	10 (0.86)	131 (1.07)	0.511
Congenital microtia	95 (0.09)	4 (0.10)	70 (0.08)	0.551	2 (0.17)	19 (0.15)	0.885

GDM, gestational diabetes mellitus; SGA, small for gestational age; LGA, large for gestational age.

Data are n (%) unless otherwise specified.

### Risk of pregnancy outcomes among younger and older women with gestational diabetes mellitus

**Table 3** shows that the associations in the binary regression analyses after adjusting for potential confounders were consistent with crude regression analyses. Among women aged 20-34 years, GDM was associated with an increased risk of all the selected maternal outcomes, preterm birth (relative risk [RR] 1.67, 95%CI 1.50–1.85), low birthweight (RR 1.24, 95%CI 1.09–1.41), LGA (RR 1.51, 95%CI 1.40–1.63), macrosomia (RR 1.54, 95%CI 1.31–1.79), and fetal distress (RR 1.56, 95%CI 1.37–1.77), as well as a decreased risk of SGA (RR 0.76, 95%CI 0.68–0.85). Among women aged 35 years or older, individuals with GDM had a higher risk of gestational hypertension (RR 2.17, 95%CI 1.65–2.83), preeclampsia (RR 2.30, 95%CI 1.81–2.93), polyhydramnios (RR 3.46, 95%CI 2.01–5.96), cesarean delivery (RR 1.18, 95%CI 1.10–1.25), preterm birth (RR 1.35, 95%CI 1.14–1.60), LGA (RR 1.40, 95%CI 1.23–1.60), macrosomia (RR 1.65, 95%CI 1.28–2.14) and fetal distress (RR 1.46, 95%CI 1.12–1.90) and were less likely to have SGA (RR 0.78, 95%CI 0.62–0.97) when compared to individuals with normal glucose level.

**Table 3:** Risk of adverse pregnancy outcomes in women with gestational diabetes mellitus, stratified by maternal age.

Outcomes	20-34 Years			35 Years or Older		
	GDM (RR, 95% CI)	Non-GDM (RR, 95% CI)	*P*-value	GDM (RR, 95% CI)	Non-GDM (RR, 95% CI)	*P-*value
Gestational hypertension						
Model 1	3.62 (3.01-4.35)	1.00	<0.001	2.43 (1.86-3.13)	1.00	<0.001
Model 2	3.27 (2.71-3.94)	1.00	<0.001	2.17 (1.65-2.83)	1.00	<0.001
Preeclampsia						
Model 1	2.78 (2.34-3.31)	1.00	<0.001	2.46 (1.95-3.11)	1.00	<0.001
Model 2	2.50 (2.09-2.98)	1.00	<0.001	2.30 (1.81-2.93)	1.00	<0.001
Placental abruption						
Model 1	2.16 (1.41-3.30)	1.00	<0.001	1.22 (0.55-2.64)	1.00	0.626
Model 2	2.08 (1.36-3.20)	1.00	<0.001	1.35 (0.61-2.97)	1.00	0.457
Placenta previa						
Model 1	1.94 (1.49-2.52)	1.00	<0.001	1.18 (0.79-1.73)	1.00	0.421
Model 2	1.87 (1.43-2.42)	1.00	<0.001	1.03 (0.68-1.53)	1.00	0.900
Polyhydramnios						
Model 1	2.25 (1.44-3.52)	1.00	<0.001	3.59 (2.11-6.08)	1.00	<0.001
Model 2	2.21 (1.41-3.47)	1.00	<0.001	3.46 (2.01-5.96)	1.00	<0.001
Cesarean delivery						
Model 1	1.49 (1.43-1.54)	1.00	<0.001	1.31 (1.25-1.37)	1.00	<0.001
Model 2	1.36 (1.30-1.42)	1.00	<0.001	1.18 (1.10-1.25)	1.00	<0.001
Preterm birth						
Model 1	1.69 (1.52-1.87)	1.00	<0.001	1.38 (1.16-1.62)	1.00	<0.001
Model 2	1.67 (1.50-1.85)	1.00	<0.001	1.35 (1.14-1.60)	1.00	<0.001
Low birthweight						
Model 1	1.27 (1.12-1.43)	1.00	<0.001	1.22 (1.00-1.48)	1.00	0.050
Model 2	1.24 (1.09-1.41)	1.00	<0.001	1.18 (0.96-1.44)	1.00	0.108
SGA						
Model 1	0.78 (0.70-0.87)	1.00	<0.001	0.79 (0.63-0.99)	1.00	0.037
Model 2	0.76 (0.68-0.85)	1.00	<0.001	0.78 (0.62-0.97)	1.00	0.030
LGA						
Model 1	1.49 (1.37-1.60)	1.00	<0.001	1.37 (1.20-1.55)	1.00	<0.001
Model 2	1.51 (1.40-1.63)	1.00	<0.001	1.40 (1.23-1.60)	1.00	<0.001
Macrosomia						
Model 1	1.52 (1.31-1.77)	1.00	<0.001	1.59 (1.23-2.05)	1.00	<0.001
Model 2	1.54 (1.31-1.79)	1.00	<0.001	1.65 (1.28-2.14)	1.00	<0.001
Fetal distress						
Model 1	1.72 (1.52-1.96)	1.00	<0.001	1.57 (1.21-2.02)	1.00	<0.001
Model 2	1.56 (1.37-1.77)	1.00	<0.001	1.46 (1.12-1.90)	1.00	0.004
Apgar score < 7 at 5 min						
Model 1	0.73 (0.46-1.15)	1.00	0.174	0.81 (0.42-1.53)	1.00	0.512
Model 2	0.75 (0.47-1.19)	1.00	0.218	0.85 (0.44-1.63)	1.00	0.625
Congenital microtia						
Model 1	1.32 (0.48-3.62)	1.00	0.584	1.11 (0.26-4.76)	1.00	0.885
Model 2	1.41 (0.51-3.87)	1.00	0.508	1.00 (0.23-4.33)	1.00	0.996

GDM, gestational diabetes mellitus; SGA, small for gestational age; LGA, large for gestational age; RR, relative risk; CI, confidence interval.

Model 1: shows crude relative risk.

Model 2: Adjusted for parity, obesity, examination at first trimester, number of prenatal visits, previous cesarean history, ART, and number of prior spontaneous or induced abortions.

### Epidemiologic interaction between gestational diabetes mellitus and advanced maternal age on adverse pregnancy outcomes

**Figure 2:**
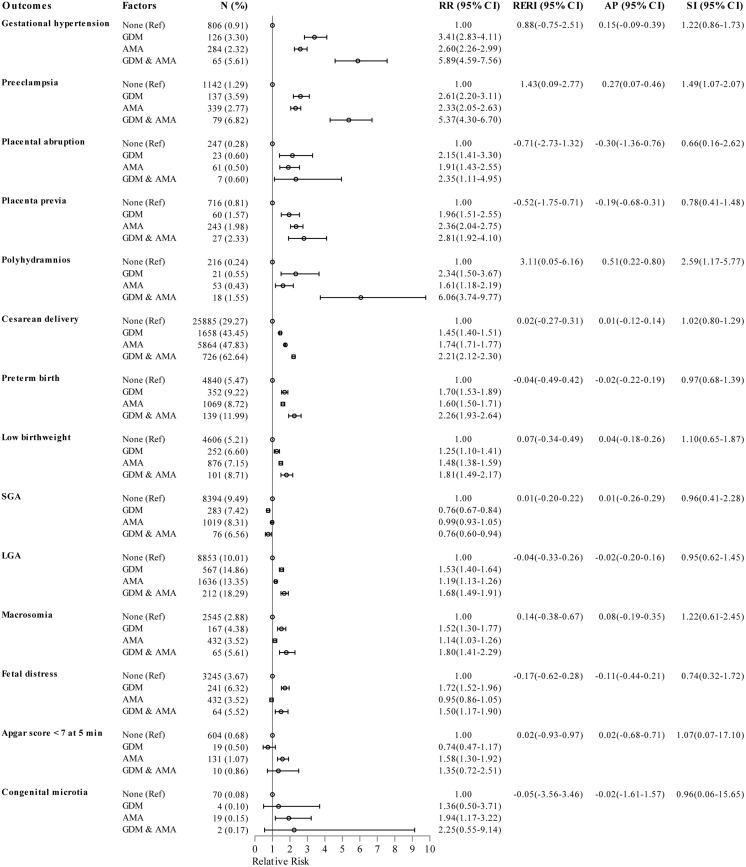
Epidemiologic interaction of gestational diabetes mellitus and advanced maternal age on the risk of pregnancy outcomes. RRs (95% CIs) were adjusted for parity, obesity, and number of prenatal visits. GDM, gestational diabetes mellitus; AMA, advanced maternal age; SGA, small for gestational age; LGA, large for gestational age; Ref, reference group; RR, relative risk; CI, confidence interval; RERI, relative excess risk due to interaction; AP, attributable proportion due to interaction; SI, synergy index.

As shown in **Figure 2**, the logistic regression models with adjustment for parity, obesity, and number of prenatal visits manifested that the RR of polyhydramnios was 2.34 for individuals with GDM only, 1.61 for individuals with AMA only, and 6.06 for individuals with both GDM and AMA when compared to those without GDM aged 20-34 years. The combined effect of GDM and AMA on polyhydramnios was markedly greater than the sum of the separate effect, with a RERI of 3.11 (95%CI 0.05-6.16), AP of 0.51 (95%CI 0.22-0.80), and SI of 2.59 (95%CI 1.17-5.77). In addition, the RR for concurrent GDM and AMA on preeclampsia was slightly higher than the sum of the individual effect, with a RERI of 1.43 (95%CI 0.09-2.77), AP of 0.27 (95%CI 0.07-0.46), and SI of 1.49 (95%CI 1.07-2.07).

## Discussion

In this study, GDM was associated with an elevated risk of gestational hypertension, preeclampsia, polyhydramnios, cesarean delivery, preterm birth, LGA, macrosomia, and fetal distress and a decreased risk of SGA in both younger and older women. Interestingly, we observed the additive interactions between GDM and AMA on the risk of polyhydramnios and preeclampsia.

GDM is associated with an increased risk of various maternal outcomes. Some evidences manifested that GDM elevated the occurrence of gestational hypertension and preeclampsia, which were in accordance with our study (7, 9, 27). As all we know, insulin resistance play a role in the pathogenesis of hypertension in pregnancy (28). Among younger women with GDM, increased risk was found for placental abruption. Hyperglycemia during pregnancy may induce a condition of placental thickening, and this constant state was associated with placental abruption (29). GDM also increased the incidence of placenta previa in younger women. The greater proportions of prior abortions, using ART, and previous cesarean history may help explain this outcome (30). However, the elevated risk of placental abruption and placenta previa was not observed in older individuals, implying that the association of GDM with the two outcomes may be modified by maternal age. In addition, a higher risk of polyhydramnios and cesarean delivery was found in patients with GDM compared to those without GDM. The findings were in accordance with studies in Ethiopia (9) and Ireland (31).

A relationship between GDM and adverse infant outcomes was also found in our study. Offspring of women with diabetes are considered to be at an elevated risk of fetal distress. In this study, the incidence of fetal distress was higher in offspring of mothers with GDM when compared to those unaffected by GDM, which was in line with the study of Zhuang et al. (10). Consistent with other studies (8, 32), the logistic regression model demonstrated that individuals with GDM had a higher risk of preterm birth than those with normal glucose tolerance. This may be explained by the higher rate of hypertension, placenta previa, and fetal distress (33, 34). We also observed a close association between GDM and the risk of developing LGA and macrosomia. Pregnant women with GDM had an over 1.4-fold risk of LGA and macrosomia compared to those without GDM. This is in accordance with studies in Germany (32), the United States (35), and Canada (36). The occurrence of these neonatal outcomes may be linked to maternal hyperglycemia and insulin resistance that subsequently resulted in fetal hyperinsulinemia and thus contributed to nutrient utilization and fetal overgrowth (37, 38). Individuals with GDM had a higher incidence of low birthweight than non-GDM counterparts. As GDM was associated with an elevated risk of preterm birth and a decreased risk of SGA, it was suggested that preterm birth rather than intrauterine growth restriction was the primary cause of low birthweight. Similar to our findings, population-based studies conducted in Taiwan (39) and mainland of China (40) indicated that GDM increased the risk of low birthweight by 64% and 37%, respectively.

This study shows that the interactions between GDM and AMA were more strongly associated with the risk of polyhydramnios than the sum of the separate effect. GDM and AMA were both independent risk factors for polyhydramnios (31, 41), however, further researches about their interactions on polyhydramnios were scarce. The causal relationship between GDM, AMA, and polyhydramnios occurrence may be explained by the following evidences. First, maternal hyperglycemia is usually accompanied by an increased level of fetal blood sugar, and this condition induces osmotic diuresis and subsequently leads to polyhydramnios (42). Second, increasing maternal age is followed by a significantly elevated concentration of human brain natriuretic peptide, where brain natriuretic peptide plays a role in the pathogenesis of polyhydramnios (43, 44). Third, AMA also significantly increases the risk of maternal hyperglycemia (45), further promoting the occurrence of polyhydramnios. In addition, a slightly additive interaction of GDM and AMA on preeclampsia incidence was uncovered. We hypothesized that the excretion of proteinuria increased with increasing age-related glomerular sclerosis (46), along with the hypertension induced by insulin-resistance, ultimately resulted in preeclampsia for women with GDM aged 35 years or older. Similarly, a registry-based study of 230 003 pregnant women in Finland indicated that combining GDM and AMA clearly had an increasing impact on preeclampsia, but the study lacked data of their interactions (11). All in all, our study provides evidences that there is a synergistic effect between GDM and AMA on polyhydramnios or preeclampsia, which may help us comprehensively estimate the health hazard of GDM and AMA.

The main strengths of our study were the large population-based register data, the maternal-age-stratified risk of adverse pregnancy outcomes, and the assessment of interactions between GDM and AMA on these outcomes. However, some limitations of this study were also present. Firstly, we did not distinguish between diet- and insulin-treated GDM. Secondly, an extremely small portion of maternal and infant outcomes data was missing during the retrospective collection. Thirdly, several confounding factors, such as maternal lifestyle and educational level, were absent and not included in the adjusted logistic model, which may affect the results of this study.

In conclusion, GDM was an independent risk factor for a wide range of adverse pregnancy outcomes. Women with GDM were more likely to have gestational hypertension, preeclampsia, polyhydramnios, cesarean delivery, preterm birth, LGA, macrosomia, and fetal distress when compared to those without GDM regardless of maternal age. More importantly, GDM and AMA may cooperate in a more than additive way in significantly elevating the risk of developing polyhydramnios and preeclampsia, which we should pay enough attention to in clinical practice. It is very necessary to prevent the occurrence of severe adverse pregnancy outcomes by strengthening prenatal care and diet or insulin treatment for women with both GDM and AMA.

## Data availability statement

The raw data supporting the conclusions of this article will be made available by the authors, without undue reservation.

## Ethics statement

The studies involving human participants were reviewed and approved by the Ethics Committee of Nanning Maternal and Child Health Hospital. Written informed consent for participation was not required for this study in accordance with the national legislation and the institutional requirements.

## Author contributions

JL, MZ, and WJ designed the study and revised the manuscript. JL, JY, LM, and YH contributed to the data collection. JL analyzed data and drafted the manuscript. All authors contributed to the article and approved the submitted version.
